# Enabling countries to manage outbreaks: statistical, operational, and contextual analysis of the early warning and response system (EWARS-csd) for dengue outbreaks

**DOI:** 10.3389/fpubh.2024.1323618

**Published:** 2024-01-19

**Authors:** Mikaela Schlesinger, Franklyn Edwin Prieto Alvarado, Milena Edith Borbón Ramos, Maquins Odhiambo Sewe, Corinne Simone Merle, Axel Kroeger, Laith Hussain-Alkhateeb

**Affiliations:** ^1^Global Health Research Group, School of Public Health and Community Medicine, Institute of Medicine, Sahlgrenska Academy, Gothenburg University, Gothenburg, Sweden; ^2^Directorate of Surveillance and Risk Analysis in Public Health, Instituto Nacional de Salud (INS) de Colombia, Bogota, Colombia; ^3^Department of Public Health and Clinical Medicine, Epidemiology and Global Health, Umeå University, Umeå, Sweden; ^4^Special Program for Research and Training in Tropical Diseases (TDR-WHO), World Health Organization, Geneva, Switzerland; ^5^Freiburg University, Center for Medicine, and Society (ZMG)/Institute of Infection Prevention, Freiburg, Germany; ^6^Population Health Research Section, King Abdullah International Medical Research Center (KAIMRC), King Saud Bin Abdulaziz University for Health Sciences (KSAU-HS), Ministry of National Guard - Health Affairs, Riyadh, Saudi Arabia

**Keywords:** outbreak prediction, outbreak response, dengue, Colombia, climate-sensitive diseases, vector-borne disease

## Abstract

**Introduction:**

Dengue is currently the fastest-spreading mosquito-borne viral illness in the world, with over half of the world's population living in areas at risk of dengue. As dengue continues to spread and become more of a health burden, it is essential to have tools that can predict when and where outbreaks might occur to better prepare vector control operations and communities' responses. One such predictive tool, the Early Warning and Response System for climate-sensitive diseases (EWARS-csd), primarily uses climatic data to alert health systems of outbreaks weeks before they occur. EWARS-csd uses the robust Distribution Lag Non-linear Model in combination with the INLA Bayesian regression framework to predict outbreaks, utilizing historical data. This study seeks to validate the tool's performance in two states of Colombia, evaluating how well the tool performed in 11 municipalities of varying dengue endemicity levels.

**Methods:**

The validation study used retrospective data with alarm indicators (mean temperature and rain sum) and an outbreak indicator (weekly hospitalizations) from 11 municipalities spanning two states in Colombia from 2015 to 2020. Calibrations of different variables were performed to find the optimal sensitivity and positive predictive value for each municipality.

**Results:**

The study demonstrated that the tool produced overall reliable early outbreak alarms. The median of the most optimal calibration for each municipality was very high: sensitivity (97%), specificity (94%), positive predictive value (75%), and negative predictive value (99%; 95% CI).

**Discussion:**

The tool worked well across all population sizes and all endemicity levels but had slightly poorer results in the highly endemic municipality at predicting non-outbreak weeks. Migration and/or socioeconomic status are factors that might impact predictive performance and should be further evaluated. Overall EWARS-csd performed very well, providing evidence that it should continue to be implemented in Colombia and other countries for outbreak prediction.

## 1 Introduction

Dengue, an infectious disease transmitted by *Aedes* mosquitoes (mainly *Aedes aegypti* and *Aedes albopictus*) is currently considered the fastest-spreading mosquito-borne disease in the world, with the incidence increasing 30-fold in the last 50 years (1, 2). Over half of the world's population live in areas at risk of dengue (3). Annually, dengue infects over 390 million people, kills over 10,000 people, and is responsible for 1.14 million disability-adjusted life years (DALYs) (2, 4). Dengue has seen a rise in cases due to climate change, human mobility, trade, and unplanned urbanization (5). Dengue's increasing transmission rate has created a large health burden on many communities, especially when outbreaks occur. There is currently no effective cure for dengue and the best way to minimize the dengue health burden is vector control measures of the *Aedes* mosquito (6).

The updated EWARS-csd tool (Early Warning and Response System for climate-sensitive diseases tool developed under the auspices of the Special Program for Research and Training in Tropical Diseases at the World Health Organization, TDR-WHO) was developed to predict dengue outbreaks before they occur to prevent potential outbreaks. The tool can utilize epidemiological, meteorological, social, and entomological variables to predict possible future dengue outbreaks (1). EWARS-csd includes interactive graphical features to improve results interpretation for users at the national (central) dashboard 1 and local (municipality) dashboard 2 levels. The EWARS-csd tool predicts disease outbreaks in time and space, allowing it to trigger vector control activities in areas of high transmission risk. In addition, it quantifies the magnitude (outbreak rate) and its certainty interval, which will have significant vector control and response implications. It employs the robust Distribution Lag Non-linear Model in combination with the integrated nested Laplace approximation (INLA) Bayesian regression framework (7).

The tool is operated through the open-access software “R” to make it accessible to users in Low- and Middle-Income Countries (LMICs). It does not require skilled users to operate it effectively. The tool was updated from EWARS to EWARS-csd (formerly EWARS+) in 2019 to improve the mathematical approach, provide descriptive data for users, predict the magnitude of disease incidence, provide confidence intervals, model all municipalities in a country together, and have fewer calibration features (8). Currently, EWARS-csd is being implemented in 17 countries (8). This includes Colombia, which is hyperendemic for dengue and experiences the highest mean dengue case fatality rate in the Americas (19 deaths per 10,000 symptomatic cases) (9). The TDR-WHO sponsored training, installation, and technical support in the implementation of EWARS-csd in certain municipalities of Colombia. Overall, Colombia's mandatory reporting of dengue cases and available case data, made it an optimal place to perform a validation study of EWARS-csd. A validation study is necessary because the previous version of EWARS was unable to generate results in Colombia due to inconsistency of disease trends caused by the seasonal effect (as in many Latin American countries) and because the tool did not perform well for municipalities of low endemicity.

The overall aim of this study was to validate whether the modernized EWARS-csd model provides reliable and operational alarm signals for dengue outbreaks in Colombia and elsewhere. Essentially, this study intended to assess the sensitivity and positive predictive value metrics for EWARS-csd for municipalities of different endemicity levels.

## 2 Materials and methods

### 2.1 Study area

**Figure 1:**
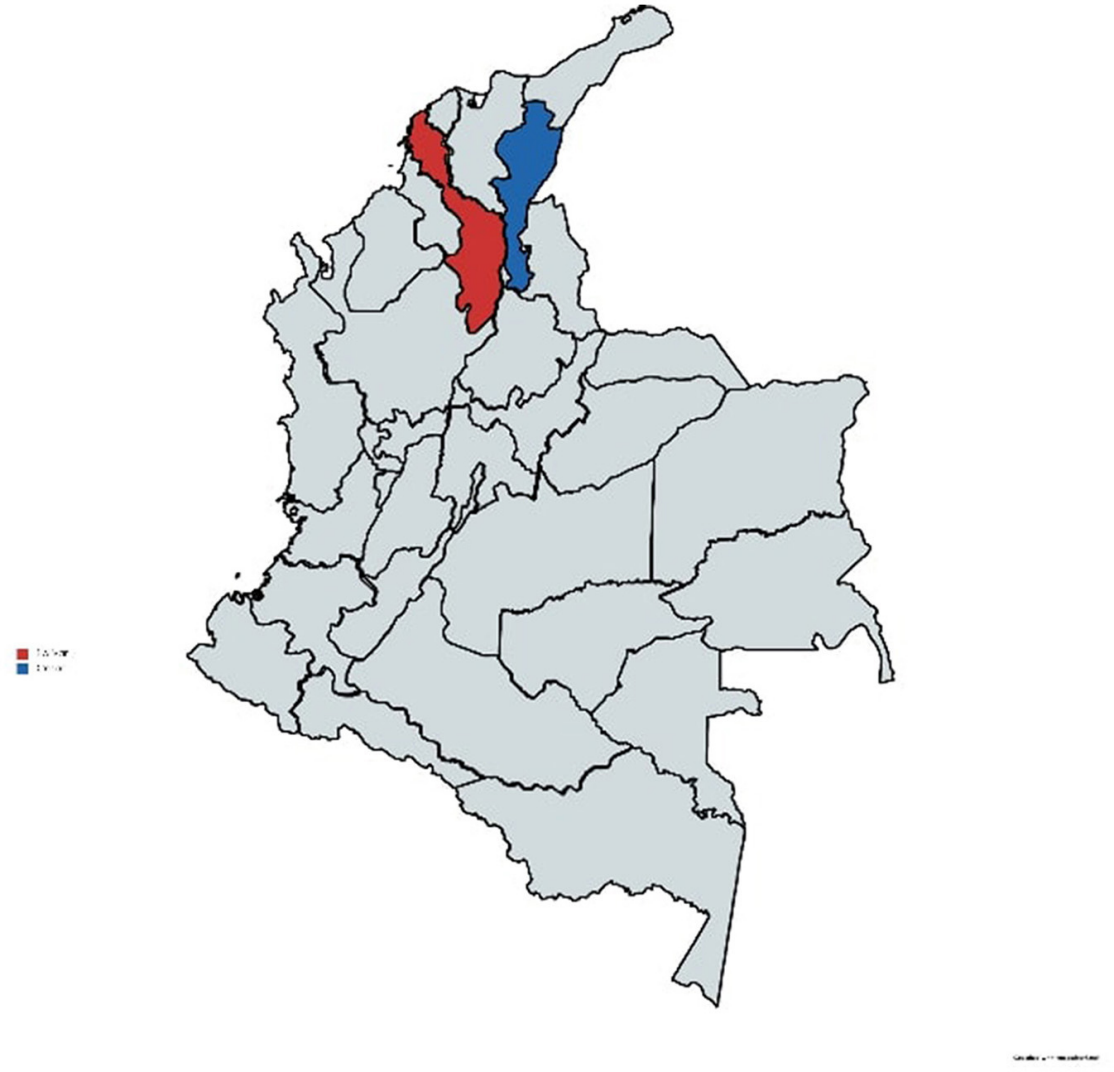
Map of the two states analyzed in the study. Bolívar (in red) and Cesar (in blue). Reprinted with MapChart.net's permission (11).

The EWARS-csd validation study was conducted in partnership with WHO, the Universities of Gothenburg and Freiburg as well the Colombian National Institute of Health's (Instituto Nacional de Salud, INS), surveillance team. For the validation study, data was used from 2 of Colombia's 32 states (“departamentos”): Bolívar and Cesar (Figure 1). Both states were part of Colombia's pilot study of EWARS-csd. The two states border each other. Cesar also shares a border with Venezuela. Bolívar has a population of 2 million, and Cesar has a population of 1.2 million (10). In total 11 municipalities of varying endemicity levels were used in this study: 4 from Bolívar and 7 from Cesar.

### 2.2 Data

Secondary data for this project was aggregated from Colombia's National Institute of Health (INS) in coordination with their national vector-control team. INS provided data on the 11 municipalities listed above, including data from 2015 to 2020 covering the mean relative humidity (%), the number of hospitalized cases, the population of the municipality, the mean temperature (°C), and the sum of rain per week (mm). The hospitalized cases data came from hospital records and were all lab-confirmed cases of dengue which required hospitalization, and these were included using the case definition according to the Ministry of Health (MoH) and INS, which was set out in the public health surveillance protocols (12). The meteorological data, which were the potential alarm indicators, was provided by the Institute of Hydrology, Meteorology and Environmental Studies (IDEAM) of Colombia. The temporal data was measured as an epidemiological week (from Sunday to Saturday) and the spatial unit was based on pre-existing administrative units (“municipios” or municipalities).

### 2.3 EWARS-csd

The EWARS-csd toolkit on the open software R was used to validate the data. Dashboard 1, which is used by the national health system level, was utilized for the validation. The tool uses spatiotemporal covariance to provide robust estimates through a distributed lag non-linear Bayesian framework (13). It uses a baseline model and non-linear function of incidence-week in order to capture seasonality or the unknown variability annually (13). The model produces out-of-sample predicted probabilities of exceeding the outbreak threshold from alarm indicator parameters. It is compared with the endemic channel, which represents the historical pattern of disease incidence or dengue hospitalization incidence.

### 2.4 Validation

The model can be tested at different calibrations to see which tool settings, such as run-in year, *z*-value (see below), and time-lag or prediction period, provide the optimal model measured by statistical metrics. In a retrospective cross-evaluation tool process, the run-in year is the year that the data would be cut between either being part of the historic data to build the model or to be part of the future data, which is used to predict outbreaks (model evaluation). The run-in years available were 2016–2020.

The *z*-value is a multiplier of the weekly standard deviation of hospitalized cases (or other outbreak indicators) (14). The importance of this calibration is to change the outbreak threshold, or the upper line of the endemic channel, which is useful to account for different endemic settings (15). *Z*-values were calibrated between 1 and 4 in this study.

The time-lag is the period between exposure (e.g., change in the climate condition) and the disease outbreak manifestation (8). This is measured in weeks. For this validation, time-lags between 8 and 14 weeks were evaluated as this range is generally supported in the literature; though, it could be expanded as the time-lag is not fully understood, especially with different variables interacting with one another (16, 17).

**Figure 2:**
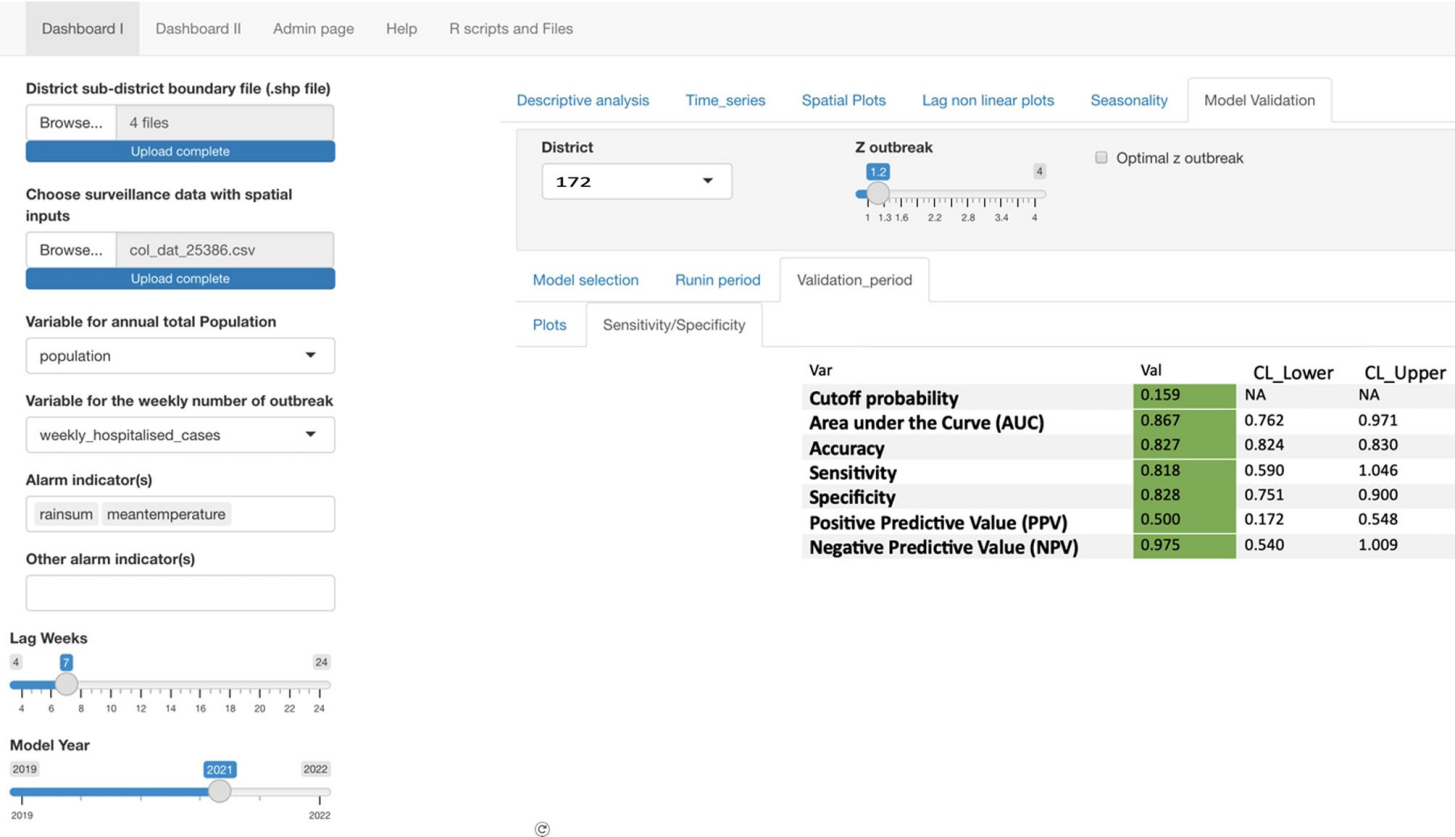
Screenshot displaying EWARS-csd dashboard 1 with different calibration variables: lag weeks, model year, and Z outbreak. It also demonstrates the outbreak variable: weekly_hospitalized_cases and alarm indicators mean temperature and rain sum. The top left indicates that it is working on the Dashboard 1 level, and the four shape files must be uploaded and the specific data from the municipalities.

To predict outbreaks, there must be defined alarm indicators, or variables which indicate that an outbreak is coming. These variables can be meteorological, entomological, or potentially social and logistic alarm indicators (15). The validation study in Colombia used the following variables as alarm indicators: rain sum (i.e., weekly rainfall) and mean weekly temperature. These variables would be used to predict the outbreak indicator. This validation study used the outbreak indicator of weekly hospitalized cases (see Figure 2 for depiction of EWARS-csd dashboard).

For the validation, optimization measurements and receiver operating characteristics (ROC) were calculated to determine the optimal calibrations of sending alarm signals. The ROC includes cutoff probability, area under the curve (AUC), accuracy, sensitivity, specificity, PPV, and NPV.

Sensitivity-the proportion of events that occurred (i.e., outbreaks) that were correctly predicted (14).

Specificity-the proportion of events that were predicted not to occur and did not occur.

PPV-the probability of following an outbreak signal by EWARS-csd that the period will truly have a disease outbreak. The proportion of true alarm signals.

NPV-the probability of following non-outbreak signals by EWARS-csd that the period will truly not have a disease outbreak. The proportion of true non-alarm signals.

### 2.5 Data calibration in EWARS-csd

For this validation study, the optimal (highest) sum of sensitivity and PPV was recorded for each municipality. To achieve this, different calibrations of the tool were evaluated, so each municipality was calculated at each unique cut-off year (2016–2020) while varying the time-lag (8–14 weeks) and then adjusting the *z*-value (1–4) to find the optimal level of sensitivity and PPV. This repetitive process allowed for an in-depth understanding of how the calibration variables interact with each other. The highest sensitivity and PPV sum for each municipality each year was then recorded as well as the other information relating to that calibration such as: the endemicity level, the cutoff probability, the AUC, accuracy, sensitivity (95% CI), specificity (95% CI), PPV (95% CI), NPV (95% CI), run-in year, *z*-value, lag-time/lag non-linear, and the sensitivity and PPV total. If multiple calibration measures resulted in the same sum of sensitivity and PPV, then the median calibration values were taken.

### 2.6 Endemicity levels

A municipality's outbreak threshold depends on the endemic channel, or the number of cases a community usually (e.g., during the past 5 years) experiences. To generate endemicity levels, interquartile ranges of the hospitalized cases were taken for all the municipalities together using Stata. Category cut-offs without “zero” cases were used to avoid the lowest range being 0. Overall, there were 966 weeks with hospitalization values of 0. Quartile <25% was considered low endemicity, quartile 25–50% was considered moderate endemicity, and quartiles >50% was considered high endemicity. The low endemicity municipalities are classified as having a median of weekly hospitalized dengue cases of 1 or less, moderate endemicity as 2 or 3, and high as over 3 hospitalizations per week.

### 2.7 Analysis

Numerical and graphical statistical descriptions were sought for each municipality and stratified by different categories. The data was used from the most optimal sensitivity and PPV per municipality. The median and range were calculated for sensitivity, specificity, PPV, NPV, run-in year, *z*-value, and the time-lag. The median was chosen because the data is not normally distributed, and the median is less sensitive to skewed data. The results were then divided by endemicity levels, provinces, and population sizes to allow for further analysis. While there are no universally agreed predefined cut-off points, this study considers optimization measurements below 50% as poor, 50–70% as fair, and above 70% as good performance results.

### 2.8 Disease incidence rates

**Figure 3:**
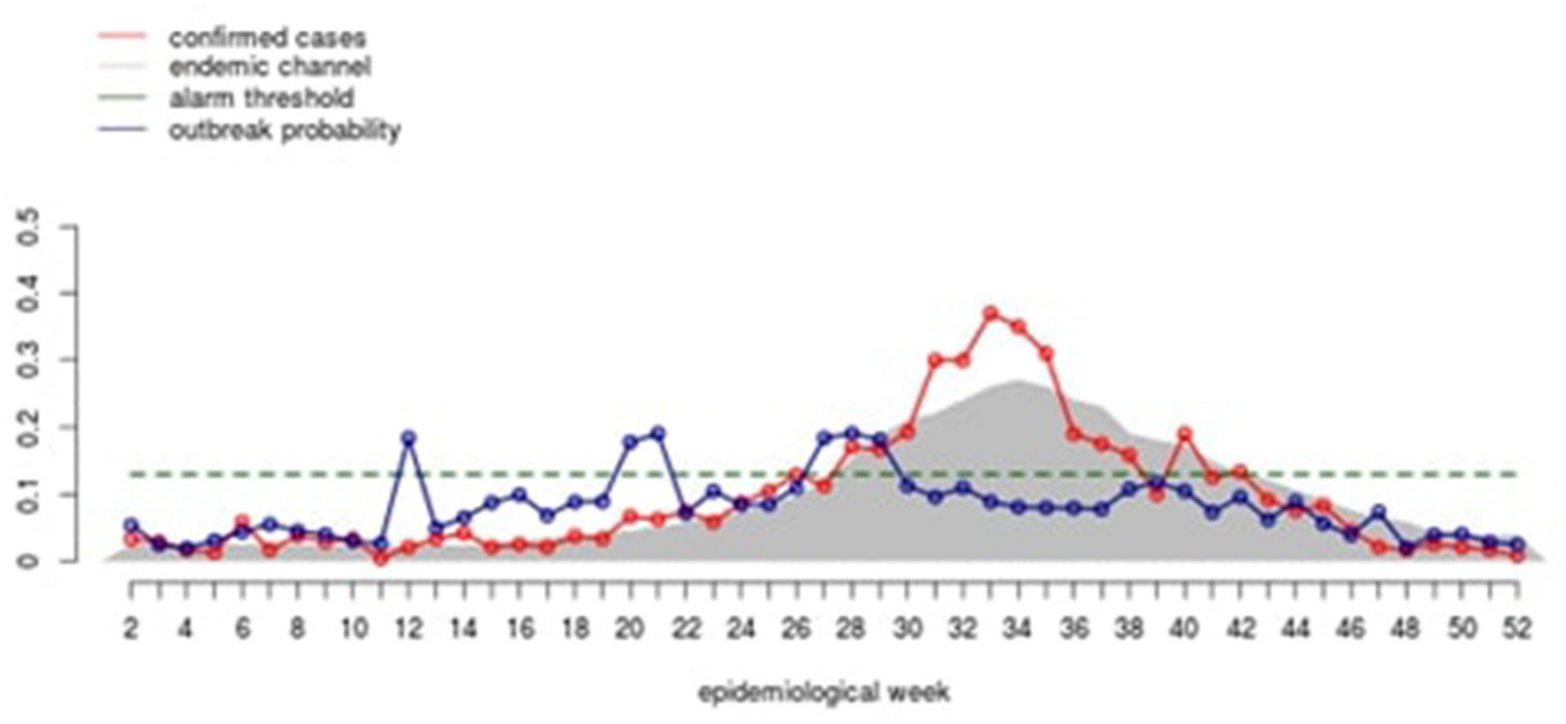
Diagram from the EWARS+ program that the municipality medical officer. The red line indicates predicted incidence, in this case hospitalized cases (the tool is currently being updated to match this heading). The blue line indicates the outbreak probability.

For further analysis, disease incidence rate graphs, computed from the corresponding municipalities, were produced to visualize how well the tool predicted outbreaks (see Figure 3). The tool predicts the magnitude of the outbreak incidence. When the exceedance probability, predicted from the alarm indicators, crosses the cutoff threshold, then it will be considered an alarm signal. The cutoff threshold is based on the endemicity of the municipality plus the standard deviation multiplied by the *z*-value. The alarm signals can be compared to when outbreaks occurred according to the disease incidence data provided.

### 2.9 Ethical considerations

This project made ethical considerations throughout the entire process. Ethical endorsement was obtained from the Ethics Committee of the University of Freiburg (N°-145/18) which was approved by local health authorities. The data validated was in agreement with and obtained from Colombia's INS. All data was taken at the aggregated level with no personal information recorded for EWARS-csd. Specific ethical approval related to the validation study was not required. The results of this study are being shared with the INS to better implement the tool for the evaluated municipalities and to prevent dengue outbreaks throughout the whole country to benefit the affected communities.

## 3 Results

### 3.1 Endemicity levels

Among the 11 municipalities studied between the years 2015 to 2020, there were 20,154 hospitalized cases of dengue. Overall, there were five low endemicity municipalities, five moderate endemicity municipalities, and one high endemicity municipality, which allows for evaluation of the tool's performance at different levels of dengue endemicity (Table 1).

**Table 1:** Median weekly dengue hospitalizations by municipality and endemicity level.

**Bolívar municipality**	**Municipality name**	**Median weekly hospitalizations**	**Endemicity level**	**Population in 2020**
170	Cartagena	3	Moderate	1,028,736
172	El Carmen De Bolívar	2	Moderate	72,595
178	Santa Cruz de Mompós	1	Low	46,408
190	Santa Rosa del Sur	1	Low	34,568
16.5-1,15.5498pt**Cesar municipality**	**Municipality name**	**Median weekly hospitalizations**	**Endemicity level**	**Population in 2020**
445	Aguachica	3	Moderate	118,652
446	Agustin Codazzi	2	Moderate	64,676
449	Bosconia	1	Low	43,326
452	Curumani	2	Moderate	39,667
464	San Alberto	1	Low	28,453
466	San Martin	1	Low	28,769
468	Valledupar	9	High	454,906

### 3.2 Summary statistics of the tool's performance

For each municipality, the highest sensitivity and PPV value is recorded in Table 2, along with the other measurements at the calibrations that resulted in the most optimal value. Municipality 172, 178, 190, 446, and 464 all had multiple calibration combinations that resulted in the same optimal sensitivity and PPV, so for these municipalities, the median value of optimal calibrations was recorded. The optimal combined sensitivity and PPV value was 2.00 in municipality 178, which was of low endemicity. The least optimal sensitivity and PPV value was 1.46 in municipality 466, also of low endemicity.

**Table 2:** Summary table of most optimal calibrations for each municipality.

**Municipality**	**Endemicity**	**Sensitivity (95% CI)**	**Specificity (95% CI)**	**PPV (95% CI)**	**NPV (95% CI)**	**Run-in year cut-off**	***Z*-value**	**Lag-time (weeks)**	**Sensitivity and PPV total**
178	Low	1.00	1.00	1.00	1.00	2020	2.45	13	2.00
190	Low	1.00	0.88	0.75	1.00	2019	3.10	10.5	1.75
449	Low	0.92	0.94	0.60	0.99	2018	4.00	11	1.52
464	Low	0.92	0.72	0.68	0.93	2018	1.25	13	1.59
466	Low	0.83	0.94	0.63	0.98	2020	3.30	9	1.46
170	Moderate	0.97	0.94	0.77	0.99	2017	2.90	11	1.74
172	Moderate	1.00	0.96	0.75	1.00	2020	1.60	13	1.75
445	Moderate	1.00	0.84	0.81	1.00	2019	1.20	12	1.81
446	Moderate	0.80	0.98	0.80	0.98	2019	3.55	13	1.60
452	Moderate	1.00	0.90	0.85	1.00	2019	1.00	13	1.85
468	High	0.92	0.57	0.67	0.88	2019	1.10	10	1.59

### 3.3 Outbreak prediction in high, middle, and low endemicity municipalities of Colombia

The tool provided disease incidence graphs of the outbreak prediction scenarios. As seen in Figure 3, the tool was fairly accurate in low endemicity municipalities as outbreak alarms were often before the outbreak points. This is indicated with the blue alarm dots that are produced when the green exceedance probability line extends beyond the red cutoff probability line. If the tool is predicting well then, the blue dots should be followed around 12 weeks later with an orange outbreak dot showing that an outbreak occurred. This graph can be summarized quantitatively.

**Figure 4:**
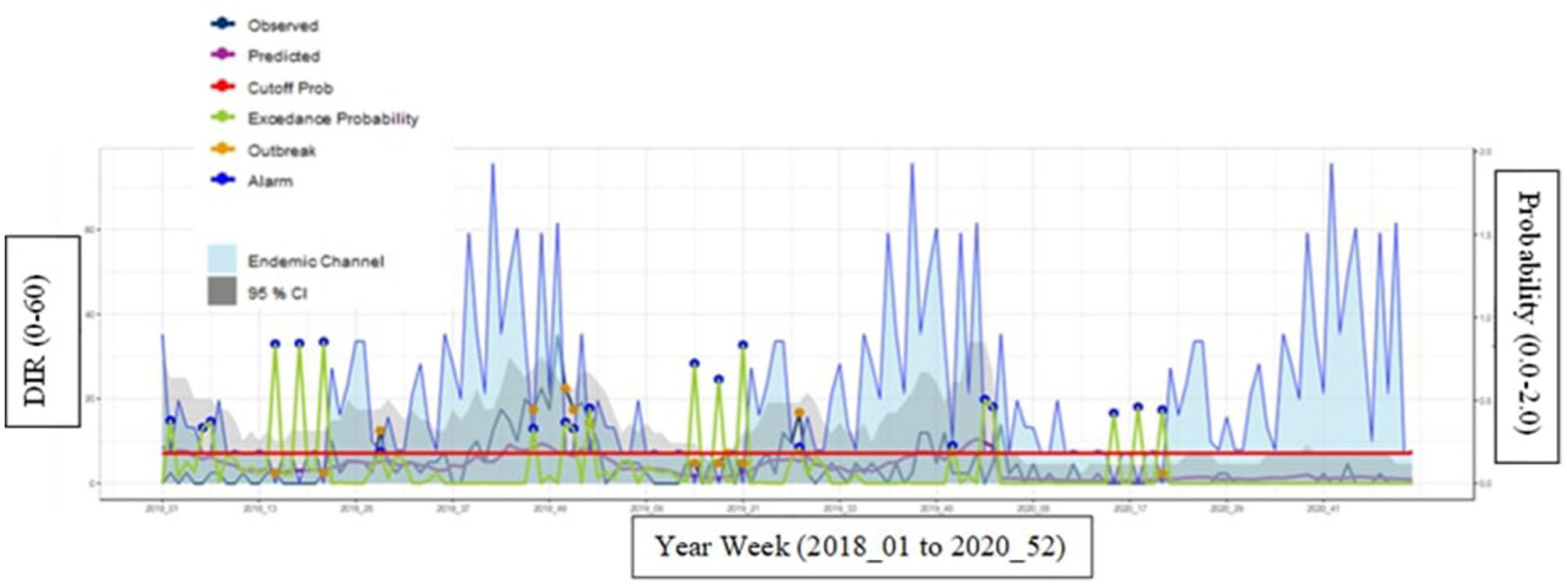
Municipality 449 (low endemicity) at most optimal calibrations with a cutoff year of 2018 and *Z*-score of 4.0. Description of how to interpret the graph. Endemic channel in light blue with 95%. Confidence intervals (gray area) representing the “normal” incidence rate of hospitalized dengue cases (upper limit = z*SD of incidence in each week). Observed incidence: Notified incidence of dengue hospitalizations. Predicted incidence: Incidence predicted by the EWARS+ tool. Cut-off for outbreak indicator (outbreak probability). When the alarm indicator (exceedance probability) crosses this line the alarm indicator turns into an alarm signal. Exceedance probability (i.e., outbreak probability): predicted weekly number of cases or incidence above the expected, i.e., above the endemic channel.

In municipality 449 (low endemicity), a good sensitivity (92%), specificity (94%), and NPV (99%) were found with a fair level of PPV (60%) (Table 2). The predicted (purple line) and observed incidence rate of hospitalized dengue cases (dark blue) lines also run quite closely to each other, indicating the tool can accurately forecast dengue incidence rates (Figure 4).

**Figure 5:**
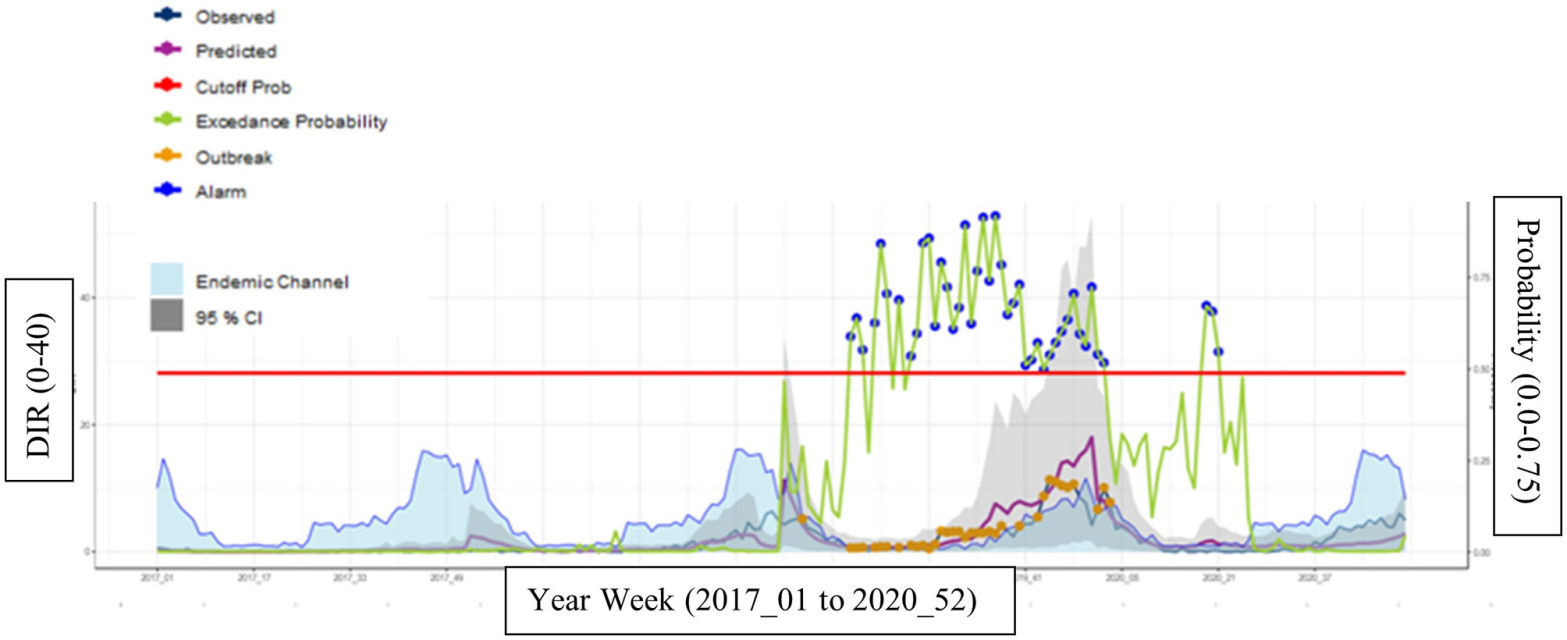
Municipality 170 (moderate endemicity) at most optimal calibrations with a cutoff year of 2017 and a *Z*-score of 2.9.

For the moderate endemic municipality 170, it also provided strong predictions as indicated by the graph (Figure 5) and Table 2, with good scores across all measurements: sensitivity (97%), specificity (94%), PPV (77%), and NPV (99%). The probability cutoff i.e., alarm threshold for municipality 170 is quite high for a moderate municipality. This is most likely because the calibration was set with years of higher dengue hospitalization incidence.

**Figure 6:**
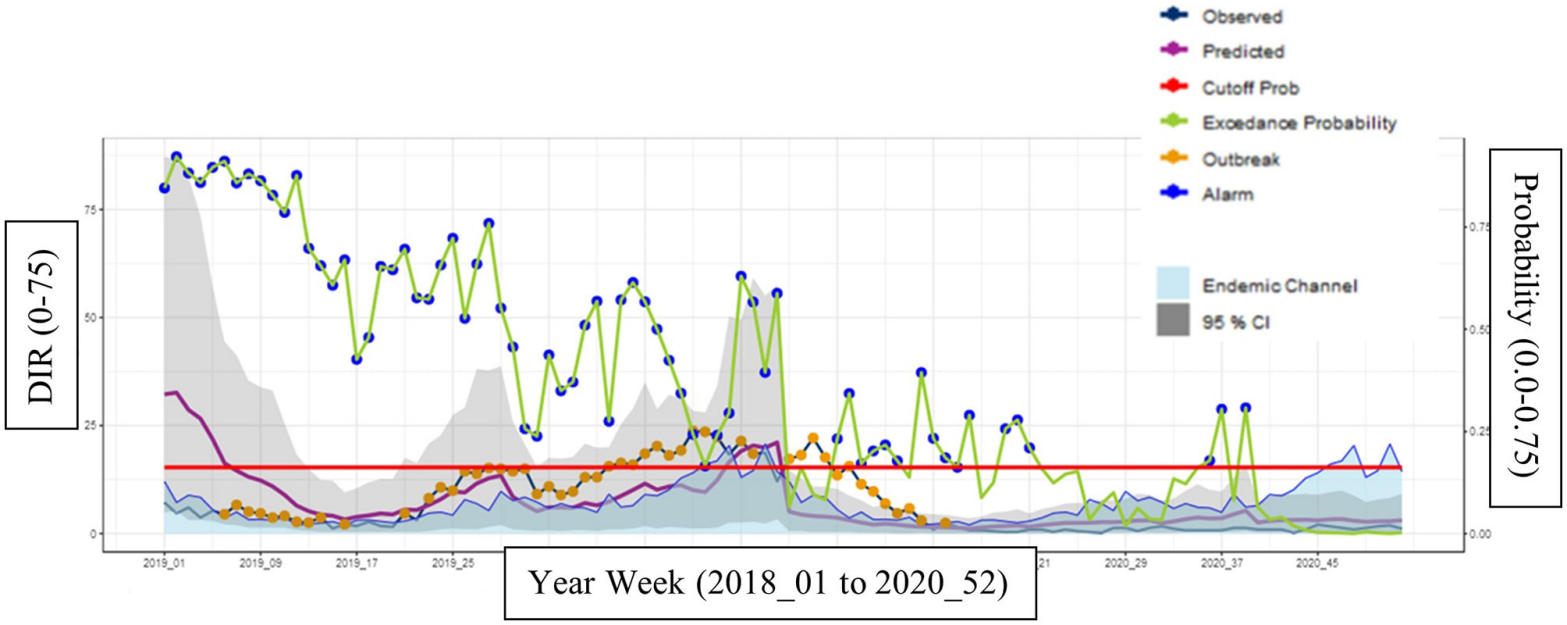
Municipality 468 (high endemicity) at most optimal calibrations with a cutoff year of 2019 and a *Z*-score of 1.1.

For the high endemic municipality 468, the disease incidence rate graphs and results provided fair predictions for specificity (57%) and PPV (67%) and good predictions for sensitivity (92%) and NPV (88%) (Figure 6 and Table 2).

### 3.4 Optimal values

When analyzing all municipalities' optimal calibrations, there were good results for the median sensitivity (0.97) with a tight range (0.80–1.00) (Table 3). It means that the model has managed to predict 97% of all outbreaks that happened that year. Specificity also had good median results (0.94) but a wider range (0.57–1.00). This means that the model managed to predict 94% of all non-outbreaks. The PPV was a lower median value (0.75) compared to the NPV (0.99), but both were still in the good range. For PPV, 75% of alarm signals for outbreaks were correctly predicting outbreaks (true positive). For NPV, 99% of the lack of alarm signals were correct in predicting an outbreak would not occur (true negative). The optimal median run-in year was 2019 and median optimal *z*-value was 2.45. The optimal median lag time was 12 (Table 3).

**Table 3:** Median and range of the most optimal sensitivity and PPV after calibration for all municipalities combined.

**Variable**	**Median (range)**
Sensitivity	0.97 (0.80–1.00)
Specificity	0.94 (0.57–1.00)
PPV	0.75 (0.60–1.00)
NPV	0.99 (0.88–1.00)
Run-in year	2019 (2017–2020)
*Z*-value	2.45 (1.00–4.00)
Lag-time (weeks)	12 (9–13)

#### 3.4.1 Endemicity levels

When endemicity was accounted for, the tool appeared optimal for moderate endemicity municipalities with sensitivity, specificity, PPV, and NPV all being highest in this level (Table 4). The tool appeared to be least optimal in the high endemicity municipality. 2019 as the cut-off year was the median value across all three municipality levels. A longer lag of 13 weeks was the median in moderate municipalities, but it was 11 and 10 weeks in low and high endemic municipalities, respectively.

**Table 4:** Median and range of the most optimal sensitivity and PPV value after calibration, separated by endemicity levels.

**Variable**	**Low endemicity**	**Moderate endemicity**	**High endemicity**
	**Median (range)**	**Median (range)**	**Median (only value)**
Sensitivity	0.92 (0.83–1.00)	1.00 (0.80–1.00)	0.92
Specificity	0.94 (0.72–1.00)	0.94 (0.84–0.98)	0.57
PPV	0.68 (0.60–1.00)	0.80 (0.75–0.85)	0.67
NPV	0.99 (0.93–1.00)	1.00 (0.98–1.00)	0.88
Run-in year	2019 (2018–2020)	2019 (2017–2020)	2019
*Z*-value	3.10 (1.25–4.00)	1.60 (1.00–3.55)	1.10
Lag-time (weeks)	11 (9–13)	13 (11–13)	10

#### 3.4.2 Differences between states

When comparing between the two states, the tool appeared to be more optimal in Bolívar with higher values of sensitivity, specificity, PPV, and NPV (Table 5). The lag-time median was the same for both at 12 weeks. The median run-in year was similar between both. The *Z*-value had a median of 2.68 in Bolívar and 1.25 in Cesar but the same range throughout.

**Table 5:** Median and range of the most optimal sensitivity and PPV value after calibration, separated by the Bolívar and Cesar provinces.

**Variable**	**Bolívar**	**Cesar**
	**Median (range)**	**Median (range)**
Sensitivity	1.00 (0.97–1.00)	0.92 (0.80–1.00)
Specificity	0.95 (0.88–1.00)	0.89 (0.57–0.98)
PPV	0.76 (0.75–1.00)	0.68 (0.60–1.00)
NPV	1.00 (0.99–1.00)	0.98 (0.88–1.00)
Run-in year	2019.5 (2017–2020)	2019 (2018–2020)
*Z*-value	2.68 (1.60–3.10)	1.25 (1.60–3.10)
Lag-time (weeks)	12 (10.5–13)	12 (9-13)

#### 3.4.3 Population effect

When separating the municipalities by population size, there were quite similar values for the performance variables between municipalities with over 100,000 people and under (Table 6). The run-in year was the same for both in 2019. The lag-time was longer at 13 weeks for smaller populations compared to 11 weeks for bigger populations.

**Table 6:** Median and range of the most optimal sensitivity and PPV value after calibration, separated by populations over and under 100,000 people per municipality.

**Variable**	**Population < 100,000**	**Population >100,000**
	**Median (range)**	**Median (range)**
Sensitivity	0.96 (0.80–1.00)	0.97 (0.92–1.00)
Specificity	0.94 (0.72–1.00)	0.84 (0.57–0.94)
PPV	0.75 (0.60–1.00)	0.77 (0.67–0.81)
NPV	0.997 (0.932–1.0)	0.99 (0.88–1.00)
Run-in year	2019 (2018–2020)	2019 (2017–2019)
*Z*-value	2.78 (1.00–4.00)	1.20 (1.10–2.90)
Lag-time (weeks)	13 (9-13)	11 (10–12)

## 4 Discussion

### 4.1 Performance of EWARS-csd

This prediction tool provided reliable results, which helped to validate its performance. The highest sensitivity (i.e., the proportion of correctly predicted outbreaks) and PPV value (i.e., the proportion of true positive alarms) were the determining factors for which calibrations to use in each municipality. When analyzing all the municipalities, the minimum value in the validity tests was 0.57 for specificity. PPV's minimum value was 0.60. For sensitivity, the minimum value was 0.80 and for NPV, it was 0.88. All values are within the predefined “fair” to “good” results category. The high sensitivity and specificity value demonstrate the tool has good predictive performance, which is important from a global health perspective to not miss outbreaks. The results also indicated that the tool showed some deficiencies regarding the proportion of true positive alarms (PPV value) but still provided fair scores of 60 and more percent. Overall, these results demonstrate the tool had lower scores in operations, compared to performance. PPV and NPV are important in the operation, or usage, of the tool as it is not effective to have an overprediction of outbreak alarms as it will not help health systems identify true outbreaks. This means that some extra resources may be deployed for outbreaks that are not likely to occur. However, because EWARS+ proposes different levels of alarm that trigger scaled-up responses, few vector control resources will be engaged if there is a false alarm that is not sustained. The tool proposes a stepwise response based on initial, early, and late alarms to balance how much action should be taken at different alarm signals; this could help to catch any false alarm signals. It is important to note that the data received did not provide municipality specific meteorological data but rather state-level data. Municipality meteorological data would grossly improve the correct prediction with a high PPV, as shown elsewhere (1). Overall, the tool performed well in both prediction and operation. The findings of this validation study are important to reconfirming outbreak prediction with a simple tool as users at the municipality level receive a simplified graph showing the alarm level (18).

The findings of this study also demonstrate successful modeling based on mean temperature and rain sum as alarm indicators (19). While other models also find associations between relative humidity and fractional cloud cover, the results of this study and its high validation scores with only two variables suggest that temperature and precipitation might be the most influential in predictions. Entomological indices like the Ovitrap index will also be particularly helpful in indicating the effort by the vector control services (20). For example, the Ovitrap index can decrease (due to vector control) the outbreak risk, despite continued high temperature or rainfall. However, a study by Ong et al. testing machine learning algorithms for dengue prediction found that meteorological variables had better predictor capabilities than vector indices, possibly because the indices measure immature mosquitoes, which cannot transmit disease (21). In addition, oftentimes vector indices data is collected inconsistently resulting in less predictive value; however, if collected consistently, entomological data has been found to have powerful predictive abilities (12). Dengue's rapid spread to new areas has caused a variety of predictive models to be developed to test a broader set of predictors in unique combinations to see if more optimal results can be obtained (see Supplementary Table 1) (22). In addition, the EWARS-csd study supports the time-lag/lag non-linear model, describing the time needed between ideal climatic conditions and dengue outbreaks, with 12 weeks being the median time with the best results (23).

### 4.2 Endemicity levels

Across all three levels of endemicity, the tool provided strong predictor signals. While there was only one highly endemic municipality to analyze (a limitation of this study), this study provided insights into how the tool works at different endemicity levels. It also supported the assumption that the tool is independent of endemicity levels. For example, the tool yielded the highest validity in moderate municipalities, though municipalities with low endemic levels also had the tool perform well. The high-endemic municipality, municipality 468-Valledupar, also performed well, but it had the lowest ROC scores across all four categories. For example, there was a noticeable low specificity value of 0.57, compared to the low and moderate municipalities having a value around 0.90. The initial hypothesis was that the tool performs better in highly endemic areas, due to more cases for run-in years and more equipped municipalities for case reporting etc. Furthermore, the vector control activities may be different in the study municipalities.

One possible reason the tool may have performed more poorly in the highly endemic municipality is because there may be more routine vector control activity already here which would have prevented the outbreaks from occurring. Retrospective data was used, and it did not contain information on vector control activities, which would be important information for future studies. Some other possible reasons for this are that Valledupar is highly populated as the capital of the Cesar municipality with over 450,000 residents. Dengue has been found to spread quicker in more populated areas (24). However, when looking at other municipalities with high populations, it did not seem to have an effect on the tool's performance. It is also possible that municipality 468's close location to the Venezuelan border and its ongoing refugee crisis impacts the dengue burden in this region in ways the tool cannot predict. Another factor could have been that the health system was overwhelmed because of the high caseloads and may have led to errors in case reporting as the low specificity indicates that it did not predict non-outbreak windows as well.

The high endemic municipality also had a shorter lag time of 10 weeks compared to the other municipalities, which would mean that vector control responses would need to be faster. Overall, all median values across the highly endemic municipality were still over the 0.50 mark, indicating the tool still performed well. Future studies should further examine how the tool works in highly endemic municipalities by employing a larger sample of municipalities and including other alarm indicators. Overall, it is promising that the tool performance is independent across endemicity levels, and it supports the idea that it can be used in places of all different endemicity, being data driven.

### 4.3 Differences between states

The 11 municipalities used in this study came from two states: Cesar and Bolívar. The two border each other and are part of the greater Atlantic Coast region of Colombia (9). They also should receive equal health funding from centralized, national resources. Though geographically close to each other, the tool performed differently between the two states. It performed better across all four measurements in Bolívar compared to Cesar. There are a variety of possible reasons why this may have occurred, and it is possible it was outside of the tool's predicting capabilities.

#### 4.3.1 Migration effect

Cesar, which is directly on the border with Venezuela, had a higher percentage of Venezuelan migrants per total population (4.3 vs. 3.8%) (25). The greater influx of migrants and refugees both living and passing through Cesar compared to Bolívar could impact Cesar's poorer performance on the EWARS-csd tool. Venezuelan migration has spread and increased arboviruses throughout Latin America and this could increase dengue's impact in Colombia (26). In 2021, 77% of Venezuelans living in Colombia lacked access to healthcare and many also suffered from food insecurity (25). These make people more prone to suffering from dengue and could increase the municipality's overall risk of dengue outbreaks. Human mobility, such as migration, is an issue that might encourage the development of a human mobility variable for EWARS-csd.

#### 4.3.2 Socioeconomic status effect

The literature on poverty's relation to dengue is mixed as poor housing infrastructure and inadequate water storage both could increase a community's risk of an outbreak (27). However, increased mobility, which may also affect those of higher SES has also been associated with outbreaks (3). In 2021, Bolívar had higher scores in human development index (0.74), health index (0.82), educational index (0.69), and income index (0.72) (28). Cesar comparatively had lower scores across all categories: human development index (0.72), health index (0.79), educational index (0.65), and income index (0.71) (28). When comparing the tool's performance between Bolívar and Cesar, Bolívar performed better across all measurements. This could suggest that the tool performs better in communities that have higher development levels as the tool does not consider sociological factors, which could also drive dengue outbreaks and response, and lead to the tool's discrepancies. While climate conditions are especially important for the tool prediction, societal influences may also impact the probability of dengue outbreaks by better informing the model of most-disadvantaged hot-spots of disease transmission. This relation should be evaluated further, and future research could explore if socioeconomic status could be a predictive measure in the tool.

#### 4.3.3 Population effect

The tool performed quite similarly for municipalities with populations over 100,000 and below. The literature often supports that mosquitoes have adapted well to urban environments (5). Some have even considered dengue an urban disease (20). However, studies still find that rural communities play an increasingly important role in dengue transmission, and studies have found that urban and rural transmission rates are similar (20). The results of this study support that the tool plays similar roles for rural and urban areas. However, when looking at the operationalization, the lag time is shorter at 11 weeks for bigger populations compared to smaller populations, which would mean that local vector control teams would have less time to respond to outbreaks. This study demonstrates that the tool works for both environments and that the population of a municipality is less important to include in the tool's optimization.

### 4.4 Limitations

One of the major limitations of this study is that the meteorological data collected from Colombia was not specific to each municipality. While Colombia has installed many local municipality meteorological stations to better analyze local conditions and support the knowledge about local climate conditions, there were administrative complications, which meant that this data was not received. Instead, the data received was homogeneous for each municipality in the same province. This resulted in the tool predicting mainly from the overall province seasonality, instead of being specific to the specific climatic conditions of the municipality. In addition, the municipalities examined within each department did not have selection criteria based on representativeness, so this is important to note for department-level analyses. Another limitation of the study is that for Bolívar's four municipalities evaluated, there was 0 mm of rainfall for each week in 2020. Although drought is a natural phenomenon which reflects a real-life scenario in Colombia during some years, the human behaviors associated with drought including how people may store water, which create hotspots for mosquitoes, may have impacted the evaluation by the model (7). For two of the four municipalities in Bolívar, 2020 as the cut-off year was most optimal, which is interesting because it has only considered temperature for the prospective data.

Another limitation of the study is that it did not consider relative humidity due to technical issues. The success of the tool's optimization without this third indicator is very promising and future studies are warranted to see if adding relative humidity could increase optimization. Also, the true number of dengue cases is unknown as many cases are not reported, so only weekly hospitalized cases were used as an outbreak indicator, which could also be underestimated due to the COVID-19 pandemic, which overlaps with the study period. This is only a small proportion of all cases, and it means less data was available to monitor dengue levels.

## 5 Conclusion

With dengue spreading around the world and its burden being felt in more communities, it is crucial that community control services are equipped with the right resources and knowledge to combat the disease. This study provides important validation of the EWARS-csd tool and specifically how it predicts dengue outbreaks in Colombia. The tool performed well across all 11 Colombian municipalities measured, across various endemicity levels and population sizes. The tool did perform slightly better in Bolívar municipalities compared to Cesar which could be due to Cesar having lower human development indexes and/or having higher migration rates from Venezuela. Colombia and the 16 other countries currently implementing the EWARS-csd tool are working to fully integrate the tool within their national surveillance program to better focus their dengue efforts on the communities most impacted. However, 128 countries are affected by dengue. This is an unfinished regional, national, and global agenda, and this study provides crucial assurance to these countries of the tool's validity. WHO has promoted EWARS-csd in predicting dengue outbreaks, and this study should provide confidence on their decision and allow them to continue expanding this tool to better prepare other communities (29).

## Data availability statement

The raw data supporting the conclusions of this article will be made available by the authors, without undue reservation.

## Author contributions

MSc: Software, Writing–original draft, Writing–review & editing, Conceptualization, Data curation, Formal analysis, Investigation, Methodology, Supervision, Validation, Visualization, Project administration, Resources. FP: Data curation, Investigation, Methodology, Validation, Writing–review & editing, Software. MB: Investigation, Data curation, Methodology, Software, Validation, Writing–review & editing. MSe: Investigation, Data curation, Methodology, Software, Validation, Writing–review & editing. CM: Software, Methodology, Writing–original draft, Conceptualization, Funding acquisition, Supervision, Writing–review & editing, Data curation, Formal analysis, Investigation, Project administration, Resources, Validation, Visualization. AK: Funding acquisition, Supervision, Writing–original draft, Writing–review & editing, Conceptualization, Methodology, Data curation, Formal analysis, Investigation, Project administration, Resources, Software, Validation, Visualization. LH-A: Funding acquisition, Methodology, Supervision, Writing–original draft, Writing–review & editing, Conceptualization, Data curation, Formal analysis, Investigation, Project administration, Software, Validation, Resources, Visualization.
