# Supplementary material for: Enabling countries to manage outbreaks: statistical, operational, and contextual analysis of the early warning and response system (EWARS-csd) for dengue outbreaks
Source: Front Public Health. 2024 Jan 19;12:1323618. doi: 10.3389/fpubh.2024.1323618 (PMC10834665; doi:10.3389/fpubh.2024.1323618)
Supplement: Supplementary file 1 [file Table_1.DOCX]

Supplementary Material

Enabling countries to manage outbreaks: Statistical, operational, and contextual analysis of the early warning and response system (EWARS-csd) for dengue outbreaks

Mikaela Schlesinger, Franklyn Edwin Prieto Alvarado, Milena Edith Borbón Ramos, Maquines Sewe, Corinne Simone Merle, Axel Kroeger, Laith Hussain-Alkhateeb^*^

*** Correspondence:** Laith Hussain-Alkhateeb: laith.hussain@gu.se

# Supplementary Figures and Tables

## Supplementary Tables

**Supplementary Table 1. Other studies that involve dengue predictive models with their methodology and variables. The bottom half displays review articles of different models.**

| **Authors** | **Methodology** | **Variables** |
| --- | --- | --- |
| Martheswaran et al. (1) | Susceptible-Infected-Removed and Bayesian Markov Chain Monte Carlo | Climatic and Seasonality Factors |
| Masrani et al. (2) | Generalized Additive Model | Climatic Factors |
| Ong et al. (3) | Machine Learning Models | Vector Indices and Climatic Factors |
| Roster et al. (4) | Machine Learning: Decision Trees, Neural Networks, SVR, and Seasonal Naïve Baseline Model | Epidemiological and Climatic Factors |
| Salim et al. (5) | Machine Learning: Support Vector Machine, Decision Trees, and Artificial Neural Networks | Climatic Factors |
| Xu et al. (6) | Structural Equation Model | Climatic Factors and Dengue Transmission Rate |
| **Reviews on Modelling Techniques** | | |
| Andraud et al. (7) | Epidemiological Mathematical Models | Serotypes |
| Hoyos et al. (8) | Machine Learning Models | Diagnostic, Epidemic and Intervention |
| Leung et al. (9) | Quantitative Models | Climatic, Demographic, and Vector Factors |
| Louis et al. (10) | Spatial and Spatiotemporal Models | Demographic, Climatic, Remote sensing, and Entomological Factors |
| Naish et al. (11) | Quantitative Models | Climatic Factors |
| Sylvestre et al. (12) | Machine Learning Models | Clinical Data, Climatic, and Big Data Factors |

References for Supplementary Table 1

1. Martheswaran TK, Hamdi H, Al-Barty A, Zaid AA, Das B. Prediction of dengue fever outbreaks using climate variability and Markov chain Monte Carlo techniques in a stochastic susceptible-infected-removed model. Sci Rep. 2022 Mar 31;12(1):5459.

2. Masrani AS, Nik Husain NR, Musa KI, Yasin AS. Prediction of Dengue Incidence in the Northeast Malaysia Based on Weather Data Using the Generalized Additive Model. Khani Jeihooni A, editor. BioMed Res Int. 2021 Oct 25;2021:1–8.

3. Ong SQ, Isawasan P, Ngesom AMM, Shahar H, Lasim AM, Nair G. Predicting dengue transmission rates by comparing different machine learning models with vector indices and meteorological data. Sci Rep. 2023 Nov 5;13(1):19129.

4. Roster K, Connaughton C, Rodrigues FA. Machine-Learning–Based Forecasting of Dengue Fever in Brazilian Cities Using Epidemiologic and Meteorological Variables. Am J Epidemiol. 2022 Sep 28;191(10):1803–12.

5. Salim NAM, Wah YB, Reeves C, Smith M, Yaacob WFW, Mudin RN, et al. Prediction of dengue outbreak in Selangor Malaysia using machine learning techniques. Sci Rep. 2021 Jan 13;11(1):939.

6. Xu Z, Bambrick H, Frentiu FD, Devine G, Yakob L, Williams G, et al. Projecting the future of dengue under climate change scenarios: Progress, uncertainties and research needs. Blacksell SD, editor. PLoS Negl Trop Dis. 2020 Mar 2;14(3):e0008118.

7. Andraud M, Hens N, Marais C, Beutels P. Dynamic Epidemiological Models for Dengue Transmission: A Systematic Review of Structural Approaches. Nishiura H, editor. PLoS ONE. 2012 Nov 6;7(11):e49085.

8. Hoyos W, Aguilar J, Toro M. Dengue models based on machine learning techniques: A systematic literature review. Artif Intell Med. 2021 Sep;119:102157.

9. Leung XY, Islam RM, Adhami M, Ilic D, McDonald L, Palawaththa S, et al. A systematic review of dengue outbreak prediction models: Current scenario and future directions. PLoS Negl Trop Dis. 2023 Feb;17(2):e0010631.

10. Louis VR, Phalkey R, Horstick O, Ratanawong P, Wilder-Smith A, Tozan Y, et al. Modeling tools for dengue risk mapping - a systematic review. Int J Health Geogr. 2014;13(1):50.

11. Naish S, Dale P, Mackenzie JS, McBride J, Mengersen K, Tong S. Climate change and dengue: a critical and systematic review of quantitative modelling approaches. BMC Infect Dis. 2014 Mar 26;14:167.

12. Sylvestre E, Joachim C, Cécilia-Joseph E, Bouzillé G, Campillo-Gimenez B, Cuggia M, et al. Data-driven methods for dengue prediction and surveillance using real-world and Big Data: A systematic review. Santos VS, editor. PLoS Negl Trop Dis. 2022 Jan 7;16(1):e0010056.
